# Prediction of Eye Colour in Scandinavians Using the EyeColour 11 (EC11) SNP Set

**DOI:** 10.3390/genes12060821

**Published:** 2021-05-27

**Authors:** Olivia Strunge Meyer, Nina Mjølsnes Salvo, Anne Kjærbye, Marianne Kjersem, Mikkel Meyer Andersen, Erik Sørensen, Henrik Ullum, Kirstin Janssen, Niels Morling, Claus Børsting, Gunn-Hege Olsen, Jeppe Dyrberg Andersen

**Affiliations:** 1 Section of Forensic Genetics, Department of Forensic Medicine, Faculty of Health and Medical Sciences, University of Copenhagen, 2100 Copenhagen, Denmark; anne.kjaerbye@sund.ku.dk (A.K.); niels.morling@sund.ku.dk (N.M.); claus.boersting@sund.ku.dk (C.B.); jeppe.dyrberg.andersen@sund.ku.dk (J.D.A.); 2 Centre for Forensic Genetics, Department of Medical Biology, UiT–The Arctic University of Norway, 9037 Tromsø, Norway; nina.mjolsnes@uit.no (N.M.S.); marianne.kjersem@uit.no (M.K.); kirstin.janssen@uit.no (K.J.); gunn-hege.olsen@uit.no (G.-H.O.); 3 Department of Mathematical Sciences, Aalborg University, 9220 Aalborg, Denmark; mikl@math.aau.dk; 4 Department of Clinical Immunology, Copenhagen University Hospital, Rigshospitalet, 2100 Copenhagen, Denmark; erik.soerensen@regionh.dk; 5 Statens Serum Institut, 2300 Copenhagen, Denmark; heul@ssi.dk

**Keywords:** forensic genetics, eye colour, rs12913832, pigmentation, DNA phenotyping

## Abstract

Description of a perpetrator’s eye colour can be an important investigative lead in a forensic case with no apparent suspects. Herein, we present 11 SNPs (Eye Colour 11-EC11) that are important for eye colour prediction and eye colour prediction models for a two-category reporting system (blue and brown) and a three-category system (blue, intermediate, and brown). The EC11 SNPs were carefully selected from 44 pigmentary variants in seven genes previously found to be associated with eye colours in 757 Europeans (Danes, Swedes, and Italians). Mathematical models using three different reporting systems: a quantitative system (PIE-score), a two-category system (blue and brown), and a three-category system (blue, intermediate, brown) were used to rank the variants. SNPs with a sufficient mean variable importance (above 0.3%) were selected for EC11. Eye colour prediction models using the EC11 SNPs were developed using leave-one-out cross-validation (LOOCV) in an independent data set of 523 Norwegian individuals. Performance of the EC11 models for the two- and three-category system was compared with models based on the IrisPlex SNPs and the most important eye colour locus, rs12913832. We also compared model performances with the IrisPlex online tool (IrisPlex Web). The EC11 eye colour prediction models performed slightly better than the IrisPlex and rs12913832 models in all reporting systems and better than the IrisPlex Web in the three-category system. Three important points to consider prior to the implementation of eye colour prediction in a forensic genetic setting are discussed: (1) the reference population, (2) the SNP set, and (3) the reporting strategy.

## 1. Introduction

Prediction of physical traits (externally visible characteristics) from DNA can be important in criminal cases with no apparent suspects. Multiple assays for forensic prediction of eye colour, hair colour, skin colour, and biogeographic ancestry have been developed with varying accuracies [1,2,3,4,5]. Prediction of biogeographic ancestry can give an indirect indication of a person’s appearance. However, for individuals of European ancestry, there are large variations in eye colour. A direct description of the eye colour of a perpetrator could aid police investigators in focusing on a smaller group of individuals. The most widely known system for eye colour prediction is the IrisPlex assay [3], which is based on six SNPs in six genes (*HERC2*, *OCA2*, *SLC24A4*, *SLC45A2*, *TYR*, and *IRF4*) that are associated with eye colour variation. The IrisPlex predicts eye colour in three categories: blue, intermediate, and brown [3,6]. It is well known that a single SNP in *HERC2*, rs12913832, is almost perfectly associated with blue and brown eye colour [7,8]. The SNP is located in the promoter region of *OCA2* and strongly associated with *OCA2* expression [9]. In 2012, Visser and co-workers demonstrated that the *OCA2* expression was increased in melanocytes carrying the rs12913832: A allele and decreased in melanocytes carrying the rs12913832: G allele. Hence, individuals carrying a rs12913832: A allele (genotypes rs12913832: AA and rs12913832:AG) are expected to have brown eye colour, and individuals carrying the genotype rs12913832:GG are expected to have blue eye colour. Eye colour prediction using the IrisPlex relies heavily on rs12913832 [10]. Thus, the IrisPlex prediction shows high accuracies for blue and brown eye colour but low accuracy for intermediate eye colour [3]. Some individuals do not follow the expected genotype–phenotype patterns of rs12913832, and for these individuals, the IrisPlex eye colour predictions are incorrect [10,11]. We previously performed in-depth sequencing analyses of individuals with incorrect IrisPlex eye colour predictions and identified variants in *OCA2*, *TYRP1*, *TYR*, and *SLC24A4* that may explain the incorrectly predicted eye colours [11,12]. In this study, we typed those variants and the IrisPlex SNPs in 757 European individuals and selected 11 SNPs for a new eye colour prediction model, named Eye Colour 11 (EC11). We modelled and tested the EC11 in an independent data set of 523 Norwegian individuals and compared the predictions with a model solely based on the rs12913832 SNP and a model based on the six IrisPlex SNPs (called IrisPlex Norway) using different reporting systems. Lastly, we compared the results with those obtained with the IrisPlex online prediction tool (IrisPlex Web) [3].

## 2. Materials and Methods

### 2.1. Samples and DNA Extraction

A total of 757 healthy individuals residing in Denmark, Sweden, or Italy comprised the variant discovery data set. The model data set included 523 healthy individuals living in Tromsø and Bodø (Norway). Blood samples were collected from employees and students at universities, residents and employees at health care centres, employees at hospitals, and through the Danish Blood Donor Study as described elsewhere [10,13,14]. DNA was extracted using the QIAamp DNA Blood Mini Kit (Qiagen, Hilden, Germany), the QIAsymphony DNA Midi Kit (Qiagen, Hilden, Germany), or the PrepFiler™ Express DNA Extraction kit (Thermo Fisher Scientific, Waltham, MA, USA) following instructions from the manufacturers. The use of material was approved by the Danish Ethical Committee (M-20090237, H-4-2009-125, and H-3-2012-023), the Ethical Committee of Azienda Ospedaliera Ospedal Sant’Anna di Como (U.0026484.23-11-2012), the Ethical Committee of the University of Milan-Bicocca (P.U. 0033373/12), and the Faculty of Health Sciences, UiT–The Arctic University of Norway (2021/2034). All participants gave signed and informed consent and all samples were anonymised.

### 2.2. Quantitative Eye Colour Measurements and Eye Colour Categorisation

A digital photograph of one of the eyes of each participant was taken as described previously [13]. The pixel index of the eye score (PIE-score) was calculated from jpeg images 639 × 426 pixels as: (number of blue pixels–number of brown pixels)/(number of blue pixels + number of brown pixels) using the Digital Iris Analysis Tool (DIAT) [13]. The PIE-scores ranged from −1 to 1; eye colour photos with a PIE-score of 1 had only blue pixels, and eye colour photos with a PIE-score of −1 had only brown pixels. Eye colour was also categorised in a two-category (blue and brown) and a three-category (blue, intermediate, and brown) system. The categorisation was based on the PIE-score and our recent study on the perception of blue and brown eye colours [15]. In the two-category system, eye colours with PIE-score > 0.2 were categorised as blue, and eye colours with PIE-score ≤ 0.2 were categorised as brown. In the three-category system, eye colours with PIE-score > 0.8 were categorised as blue, eye colours with PIE-score ≤ 0.8 and ≥−0.5 were categorised as intermediate, and eye colours with PIE-score < −0.5 were categorised as brown. The number of individuals in each eye colour category and the mean PIE-score in the discovery data set and the model data set are shown in Table 1.

### 2.3. Variant Typing (Discovery Data Set)

A total of 44 pigmentary variants were investigated. The variants included all six IrisPlex SNPs [3], five variants in *OCA2* found to be associated with blue eye colour in individuals with the rs12913832:GA genotype [11], as well as 33 variants in *IRF4*, *TYRP1*, *SLC24A4*, and *TYR*, associated with brown eye colour in individuals with the rs12913832: GG genotype [12]. We included three variants in *TYRP1* (rs201447946, rs74606098, and rs79586719, r^2^ ≥ 0.91) to tag one large haploblock associated with eye colour [12]. Two variants in *TYR* were also in strong LD (rs1126809 and rs1393350, r^2^ = 0.92). rs1393350 was part of the IrisPlex [3], and rs1126809 was found to be of importance in individuals with brown eye colour [12]. The 757 individuals in the variant discovery data set were previously typed for the IrisPlex SNPs and the five *OCA2* variants [10,11]. The 33 variants in *IRF4*, *TYRP1*, *SLC24A4*, and *TYR* were typed using two multiplexes, a 24 plex and an 11 plex, respectively (Table S1). The 24 plex included rs12913832 and 23 variants in *SLC24A4* and *TYRP1* (rs10131374, rs12590749, rs12880508, rs12894551, rs17128288, rs17128324, rs34755843, rs35617057, rs4904887, rs4904891, rs4904897, rs4904927, rs59977926, rs7144273, rs7152962, rs7401792, rs10491745, rs1408799, rs201447946, rs62538950, rs62538956, rs74606098, and rs79586719). The 11 plex comprised rs1393350 from the IrisPlex and 10 variants in *IRF4* and *TYR* (rs1393350, rs1050976, rs10530949, rs12211228, rs9378807, rs11018509, rs1126809, rs2047512, rs34749698, rs7120151, and rs9919559). Samples were typed with the iPLEX™ Assay (Agena Bioscience, Hamburg, Germany) and analysed with matrix-assisted laser desorption-ionization time of flight mass spectrometry (MALDI-TOF MS) using the MassARRAY Analyzer 4 System (Agena Bioscience, Hamburg, Germany). The PCRs contained 0.5 µL 10X PCR buffer, 0.4 µL MgCl2 (25 mM), 0.1 µL dNTP mix (25 mM), 0.2 μL PCR Enzyme, 1 μL primer mix (1 µM of each primer), and 1 µL DNA (≥1 ng). Thermal cycling comprised initial denaturation for 2 min at 94 °C followed by 45 cycles of denaturation at 94 °C for 20 s, annealing at 56–58 °C for 30 s (56 °C for the 11 plex and 58 °C for the 24 plex), and extension at 72 °C for 1 min. A final extension step at 72 °C for 3 min was included. Shrimp alkaline phosphate (SAP) treatment, single-base extension (SBE) reactions, and preparation for mass analysis was carried out following the manufacturer’s recommendations. In the MassARRAY Nanodispenser RS1000, 5–15 nl sample was robotically spotted onto a SpectroCHIP^®^ II (Agena Bioscience, Hamburg, Germany). Mass analysis was carried out using the MassARRAY Analyzer 4, and the mass spectra were analysed with the Typer 4.0 software (Agena Bioscience, Hamburg, Germany). The Hardy–Weinberg equilibrium (HWE) and pairwise r^2^ values for linkage disequilibrium (LD) were calculated with HaploView version 4.2 [16].

### 2.4. Selection of Variants for Eye Colour Prediction Model (Discovery Data Set)

Eye colour was considered in three different reporting systems, the quantitative system (PIE-score), the two-category system, and the three-category system. For numerical reasons (statistical modelling and stability), the PIE-score (values from −1 to 1) in the quantitative system was transformed to resemble unbounded values. The transformation had an inverse, such that any real number could be transformed into a PIE-score. The PIE-score, r, was transformed by:

y = f(r) = logit (0.5 + r*0.499)

Genotypes were coded as 0, 1, or 2 according to the number of minor alleles, where the minor alleles were determined based on the allele frequencies in all samples. Due to low allele frequency, the two variants rs121918166 and rs74653330 were combined [11]. rs12913832 was considered dominant with AA and AG as 0 and GG as 1. For stability, the discovery data set (757 individuals) was randomly divided into a training set (2/3) and a test set (1/3). This was repeated 100 times. If a training set resulted in fixed variants (i.e., only one genotype observed at a base position), a new set was randomly selected. In the quantitative system (transformed PIE-score) and in the two-category system, data were analysed with three different mathematical models: (i) LASSO model with main effects [17,18]; (ii) LASSO model (with the distribution family depending on the system; Gaussian for the quantitative system and binomial for the two-category system) with main effects and all pairwise interactions between rs12913832 and all other variants (still obeying the hierarchical principle such that the main effects must also be included); and (iii) a regression tree [19]. In the three-category system, data were analysed with a classification tree [19]. All seven mathematical models were fitted on the training set, which was also used to compute variable importance of the top 20 variables (variants). The test data were used for testing and estimating the test error. For LASSO regressions, the variables were standardised (such that all had a standard deviation of (1)), and the tuning parameter was chosen by cross-validation with 10 folds to get the most regularised model with an error within one standard error of the minimum error amongst the folds. For LASSO regressions, the absolute value of the estimated effect was used as variable importance. For regression and classification trees, the variable importances provided by the R package *rpart* version 4.1–15 were used [19]. Variable importances were standardised by dividing the importance of each variant with the sum of importances within each model. The mean variable importance (across all models) was used to rank variables (i.e., the variants). The top 12 performing variants were selected for an eye colour prediction model.

### 2.5. Variant Typing (Model Data Set)

The 12 selected variants and two non-selected SNPs from the IrisPlex [3] were typed with SNaPshot (rs12913832, rs12896399, rs16891982, rs1800407, rs10131374, rs1126809, rs121918166, rs1408799, rs1800401, rs4904927, rs7120151, rs74653330, rs12203592, and rs1393350) in the model data set comprising 523 Norwegian individuals (Table S2). DNA was amplified in one multiplex reaction (14-plex) using the Qiagen Multiplex PCR kit (Qiagen, Hilden, Germany) in a final reaction volume of 10 µL. The PCR comprised denaturation for 15 min at 95 °C followed by 35 cycles of 94 °C for 30 s, 58 °C for 30 s, 72 °C for 30 s, and a final extension step of 72 °C for 10 min. A total of 5 µL amplified DNA/PCR product was treated with 2 µL ExoSAP-IT™ (Thermo Fisher Scientific, Waltham, MA, USA) for 60 min at 37 °C and 15 min at 75 °C. Single-base extension was performed with 1 µL cleaned PCR product, 2 µL SNaPshot™ Multiplex Ready Reaction Mix, 1 µL nuclease-free water (G-Biosciences), and 1 µL SBE primer mix (Table S2). Thermal cycling comprised 30 cycles of 96 °C for 10 s, 55 °C for 5 s, and 60 °C for 30 s. SBE products were treated with 1 µL SAP (Thermo Fisher Scientific, Waltham, MA, USA) for 60 min at 37 °C and 15 min at 75 °C. Separation and detection of SBE products were carried out on an ABI 3500 Genetic Analyser (Thermo Fisher Scientific, Waltham, MA, USA) using the FragmentAnalysis36_POPxl run module (POP-4™ polymer, 36 cm capillary, Dye Set E5). Capillary electrophoresis was performed with 1 µL SAP-treated SBE products and 20 µL Hi-Di formamide mixed with GeneScan™-120 LIZ^®^ Size Standard (200:1). The results were analysed with GeneMapper^®^ ID-X v.1.5 (Thermo Fisher Scientific, Waltham, MA, USA). One of the selected variants, rs7120151, could not be typed and was excluded. Hence, we used 11 selected variants for the eye colour prediction model (EC11).

### 2.6. Eye Colour Prediction Modelling (Model Data Set)

Eye colour prediction models were modelled with leave-one-out cross-validation (LOOCV) using the model data set (523 Norwegian individuals) and the selected variants. For observation number *i*, all observations except number *i* were used to train the model. The model was used to predict the eye colour of observation *i*, and the predicted and observed values were compared. Three different reporting systems were used (quantitative system, two-category system, and three-category system), and thereby three different ways of measuring the prediction error were employed. For the quantitative system, a linear regression model was used where the prediction error was the mean squared error. For the two-category system, a logistic regression model was used where blue was chosen as 1 and brown as 0 (without loss of generality). The prediction error for a predicted probability (p) was log(p) if the true eye colour was blue, and log(1-p) if the true eye colour was brown. For the three-category system, a multinomial logistic regression model was used [20]. The prediction error was the Kullback–Leibler divergence between the observed distribution (the observed eye colour has probability 1, and the other two categories probability 0) and the estimated probabilities. All prediction errors were out-of-sample prediction errors. For each reporting system, the modelling was performed with three different variant (SNP) sets: the six IrisPlex SNPs, the 11 SNPs selected in this study (EC11), and rs12913832 alone. This resulted in a total of nine models. Lastly, we used the IrisPlex online tool (model called IrisPlex Web) (https://hirisplex.erasmusmc.nl/, accessed 1 July 2020) to predict eye colour in three categories.

## 3. Results

### 3.1. Allele Frequencies of 44 Variants in the Discovery Data Set

We investigated 44 pigmentary variants in our discovery data set of 757 individuals. In this work, we typed 33 variants [12] in two multiplexes using single-base extension. Eleven variants were typed in previous studies [11,13]. The allele frequencies of the 44 variants are shown in Table 2. The allele frequencies were similar in the Danish, Swedish, and Italian populations with small discrepancies between the Scandinavian (Danish and Swedish) and Italian populations, especially in rs12913832 and rs1800407 (Table S3). Moreover, two variants, rs12913832 and rs16891982, deviated significantly from the Hardy–Weinberg equilibrium (HWE) (*p*-value < 0.001) in the discovery data set. This could be explained by positive selection in the European population or a lack of random mating between the Scandinavian (Danish and Swedish) and the Italian populations.

### 3.2. Selection of Variants for Eye Colour Prediction (Discovery Data Set)

The discovery data set with information on eye colour and the 44 pigmentary variants was analysed with seven different mathematical models. Variants were ranked according to the mean variable importance across the mathematical models (Table 3 and Table S4). rs12913832 was the top-performing SNP in all seven mathematical models and ranked number one (Table 3). The mean variable importance for rs12913832 was 74.6% (Table 3). rs121918166 and rs74653330 were combined as one variable and ranked second (Table 3). We saw a drop in mean variable importance after rank 11 (Table S4). Hence, we selected the top 11 performing variables (comprising 12 variants) for a new eye colour SNP set (Table 3). These variants had mean variable importances of at least 0.3% (Table 3 and Table S4). Four of six variants in the IrisPlex assay [3] were among the selected variants (rs12913832, rs16891982, rs1800407, and rs12896399), whereas the *TYR* variant rs1393350 (ranked 18) and the *IRF4* variant rs12203592 (not in top 20) from the IrisPlex were not selected.

### 3.3. Typing of Selected Variants and IrisPlex SNPs (Model Data Set)

The 12 selected variants (SNPs) were typed with single-base extension in an independent data set, the model data set of 523 Norwegians (bold in Table 2 and Table 3). To enable comparison with the IrisPlex, we included two SNPs from the IrisPlex assay, rs12203592 and rs1393350, in a multiplex comprising 14 SNPs. One SNP, rs7120151, that was ranked as number 9 (Table 3), was excluded due to poor amplification of the A allele. We obtained 523 complete profiles, including the 11 selected SNPs (EC11) (Table 2) and the two additional IrisPlex SNPs.

### 3.4. Eye Colour Prediction Models with EC11, IrisPlex SNPs, and rs12913832

We constructed nine different eye colour prediction models based on the model data set with 523 Norwegians by using three different reporting systems and three different SNP sets (Table 2 and Table 3). We also used the IrisPlex Web for eye colour prediction using the three-category system. Prediction errors for each eye colour prediction model are presented in Table 4. Since we used three different reporting systems, the models within each system have their own measure of prediction error. Therefore, the performance of SNP sets (including prediction errors) can only be directly compared within the same but not across different reporting systems. The EC11 models had the smallest error under all reporting systems, followed by IrisPlex Norway and rs12913832. Under the three-category system, the IrisPlex Web resulted in the highest prediction error (Table 4).

The sensitivity and specificity of eye colour prediction models in the two-category and the three-category reporting systems were determined without applying a probability threshold (pmax) (Table 5 and Table 6). Hence, the predicted eye colour was the eye colour with the highest probability value. The rs12913832 and IrisPlex Norway models showed the same sensitivity and specificity (0.92 and 0.84, respectively) in the two-category system. The EC11 was slightly more sensitive (0.96), and in turn, slightly less specific (0.82) (Table 5 and Table S5).

In the three-category system, the sensitivity was highest for blue and brown eye colours and lowest for intermediate eye colour with all three SNP sets and the IrisPlex Web (Table 6). The rs12913832 and IrisPlex Web predictions resulted in the highest sensitivity for brown eye colour: 0.95, whereas the IrisPlex Norway and EC11 models had slightly lower sensitivities (Table 6). For blue eye colour, the sensitivities were similar for all models. No individuals were predicted to have intermediate eye colour with either rs12913832 or the IrisPlex Web. Hence, the sensitivity was 0, and the specificity was 1 (Table 6). Of the individuals with intermediate eye colours, 69% and 72% were incorrectly predicted to have blue eye colours with the two models, respectively (Table S6). In contrast, intermediate eye colour predictions were obtained with IrisPlex Norway and EC11. However, only 48% and 46% of the predictions were correct (Table S6). Thus, the sensitivity was low (0.10 and 0.15, respectively), and the specificity was high (0.97 and 0.95, respectively) (Table 6).

**Figure 1:**
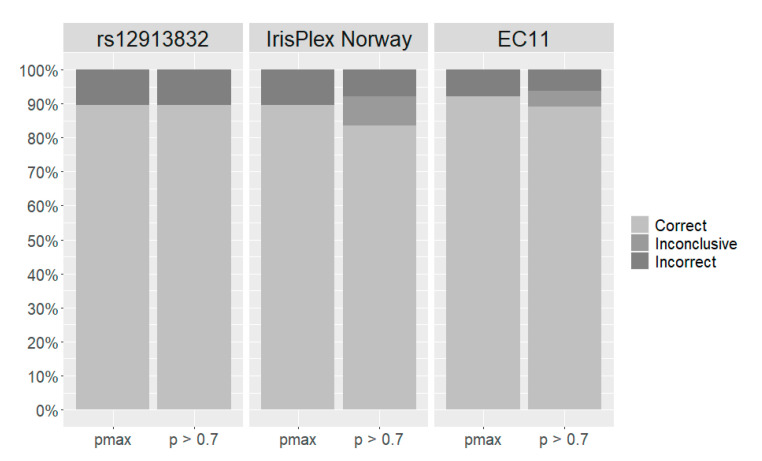
Performance of eye colour prediction models in the two-category reporting system modelled with three SNP sets: rs12913832, IrisPlex Norway, and EC11. Bars represent the percentage of correct, incorrect, and inconclusive predictions with no probability threshold (pmax) and a probability threshold of 0.7 (p > 0.7).

Figure 1 shows the percentages of correct, incorrect, and inconclusive predictions for prediction models in the two-category reporting system. Using pmax, 89% of the predictions with both rs12913832 and IrisPlex Norway were correct (Figure 1, Table S5). The EC11 model resulted in 92% correct predictions (Figure 1). Here, 93% of the blue eye colour predictions and 90% of the brown eye colour predictions were correct (Table S5). We also evaluated the prediction with a probability threshold of 0.7 in the two-category reporting system (Figure 1). If the highest prediction value was below 0.7, the prediction was defined as inconclusive. No eye colours were inconclusive with rs12913832 (Figure 1). The IrisPlex Norway and EC11 resulted in 9% and 5% inconclusive predictions, respectively (Figure 1). With the IrisPlex Norway, 62% of the inconclusive eye colours were brown according to the PIE score. With EC11, it was only 46% (Table S5).

**Figure 2:**
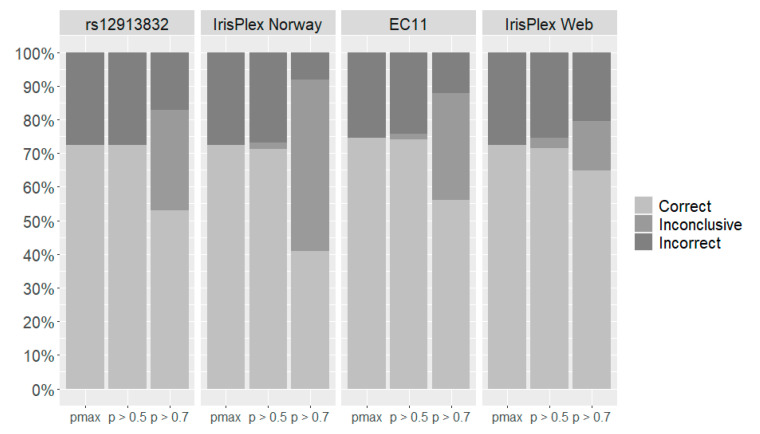
Performance of eye colour prediction models in the three-category reporting system modelled with three SNP sets: rs12913832, IrisPlex Norway and EC11, as well as performance of the IrisPlex Web prediction model. Bars represent the percentage of correct, incorrect, and inconclusive predictions with no probability threshold (pmax), probability threshold of 0.5 (p > 0.5), and probability threshold of 0.7 (p > 0.7).

In the three-category reporting system, we tested the prediction with pmax, a probability threshold of 0.5, and a probability threshold of 0.7 (Figure 2). With no probability threshold, the rs12913832, IrisPlex Norway, and IrisPlex Web models all resulted in 72% correct predictions (Figure 2, Table S6). The EC11 model resulted in a slightly higher number of correct predictions (75%) (Figure 2, Table S6). When applying a probability threshold of 0.5, predictions with rs12913832 were unchanged compared with predictions without probability threshold (Figure 2). Predictions with the IrisPlex Web tool were also similar though 3% of the total predictions were inconclusive. There was a slight decrease in the number of correct and incorrect predictions with EC11 and IrisPlex Norway as both models resulted in 2% inconclusive predictions (Figure 2). When applying a probability threshold of 0.7, blue eye colour was correctly predicted in 95% of the blue-eyed individuals with rs12913832, but no individuals were predicted to have brown eye colours (Table S6). Thus, the total number of correct predictions with rs12913832 was only 53%. The number of correct predictions using the 0.7 probability threshold was highest with IrisPlex Web (Figure 2). However, the IrisPlex Web also resulted in the highest percentage of incorrect predictions (20%) and resulted in 15% inconclusive predictions (Figure 2). The IrisPlex Norway model resulted in only 8% incorrect predictions but a high number of inconclusive predictions (51%) (Figure 2). Prediction with EC11 resulted in 12% incorrect predictions, 32% inconclusive predictions, and only 54% correct predictions (Figure 2). Of the 32% inconclusive eye colour predictions, 49% had blue eye colour, 39% had intermediate eye colour, and only 12% had brown eye colour based on the PIE-score (Table S6). Especially the percentage of brown-eyed individuals with inconclusive predictions was much lower than compared with rs12913832, IrisPlex Norway, and IrisPlex Web. For these models, the percentages were 32–65% (Table S6).

## 4. Discussion

In this study, we selected 11 SNPs (EC11) for eye colour prediction and developed new eye colour prediction models for a two-category and a three-category system that performed better than the corresponding IrisPlex and rs12913832 prediction models. The 11 SNPs in EC11 were selected from a group of 44 pigmentary variants that were originally identified in eye colour association studies [3,6] and from detailed sequence analyses of individuals with eye colours that deviated from the expected eye colour based on the genotype of rs12913832 [11,12]. The 44 variants were typed in 757 Europeans whose eye colours were quantitatively determined. Seven different mathematical models were used to rank the variants according to informativeness, and all variants with more than 0.3% mean variable importance were selected. Four of the six SNPs in the IrisPlex assay [3] were included in EC11. However, the *TYR* SNP, rs1393350 (ranked 18), was replaced by another *TYR* SNP, rs1126809 (ranked 8), and the *IRF4* gene represented by the SNP rs12203592 (not in top 20) from the IrisPlex, was not included in EC11. For the selection of SNPs, we combined individuals of Scandinavian (Danish and Swedish) and Italian descent and treated these as one population. We are aware that we may had selected different SNPs if the selection was performed solely on either the Scandinavian or the South European population. We typed the EC11 SNPs and the two additional IrisPlex SNPs in an independent data set of 523 Norwegians whose eye colours were determined with the same quantitative method as the 757 individuals in the discovery data set. We modelled nine different eye colour prediction models on the Norwegian population using the LOOCV method. Each eye colour prediction model consisted of a combination of one of three reporting systems: the quantitative system (prediction of PIE score), the two-category system (blue and brown), and the three-category system (blue, intermediate, brown), and one of three SNP sets: EC11, IrisPlex SNPs, and rs12913832. We also evaluated the IrisPlex Web model for prediction of eye colour in three categories. Based on the analysis of error rates, sensitivity, and specificity of the different eye colour prediction models, there are three main points to consider prior to implementation of eye colour prediction in a forensic genetic setting: (1) the reference population, (2) the SNP set, and (3) the reporting strategy.

### 4.1. The Reference Population

The rs12913832, IrisPlex Norway, and IrisPlex Web models showed almost identical results in the three-category system (Figure 2, Table 6). Nevertheless, a detailed comparison between the three-category IrisPlex Norway and the IrisPlex Web models highlights the importance of the reference population. The two models were based on the exact same SNPs. However, the IrisPlex Norway model was developed on the Norwegian population, which was the intended target population, whereas the IrisPlex Web model was developed on 9466 individuals of primarily European descent [6,21,22]. The two models resulted in the same number of correct predictions (72%) when no probability threshold was applied. With probability thresholds, inconclusive results were possible, and the IrisPlex Web model resulted in a higher number of correct predictions than the IrisPlex Norway model (Figure 2). However, when applying the recommended threshold for the IrisPlex Web model (p > 0.7) [21], the number of incorrect predictions was also higher (Figure 2). The two models showed similar sensitivities and specificities for blue and brown eye colour but differed for the intermediate eye colour category (Table 6). A total of 123 Norwegian individuals had intermediate eye colours according to the PIE-score (Table 1). Intermediate eye colour predictions were obtained with the IrisPlex Norway model using both pmax and p > 0.5 (Table S6), whereas no individuals were predicted to have intermediate eye colours with the IrisPlex Web model (Table S6). Hence, the IrisPlex Norway model showed an overall lower prediction error than the IrisPlex Web model (Table 4), and this emphasises the importance of modelling a prediction model on the appropriate reference population. This is in agreement with previous evaluations of the IrisPlex model which showed that prediction models based on the intended target population (the reference population) performed better than the IrisPlex Web tool [23,24]. However, it is important to note that the differences between the IrisPlex Web and the IrisPlex Norway models may not only be due to the different reference populations. Different strategies on phenotyping and categorisation of eye colour may also have contributed here. In our study, we determined the eye colour quantitatively, and the eye colour prediction models were modelled accordingly. For the IrisPlex Web model, the eye colour was evaluated by a medical researcher who categorised eye colours in blue, brown, and non-blue/non-brown (called intermediate) categories [3,6,21,22].

### 4.2. The SNP Set

The eye colour prediction models based on EC11 had the lowest prediction error rates in all three reporting systems and consistently performed better than the rs12913832, IrisPlex Norway, and IrisPlex Web models in the Norwegian population (Table 4). In the three-category reporting system, only EC11 and IrisPlex Norway were able to predict intermediate eye colours (Table 6, Table S6). For the EC11 model, intermediate eye colour predictions were even obtained with a probability threshold of 0.7 (Table S6). We did expect the prediction errors to decrease when the number of loci increased. However, the prediction errors obtained with EC11 (11 SNPs) were only slightly smaller than with IrisPlex Norway (six SNPs), closely followed by rs12913832 (one SNP) and IrisPlex Web (six SNPs) (Table 4). This shows that a single SNP, rs12913238, may be sufficient for prediction of eye colour. This SNP was ranked as number one across all mathematical models with a mean variable importance of 74.6% (Table 3). We modelled rs12913832 in a dominant matter and acknowledge that it is unreasonable to predict eye colour in three categories with a predictor variable containing only two levels (AA/AG and GG). However, even in the three-category reporting system, prediction with rs12913832 showed a lower prediction error than prediction with the IrisPlex Web (Table 4). In both the three-category reporting system and the two-category reporting system, prediction with rs12913832 and IrisPlex Norway performed almost identically (Figure 1, Figure 2). This stresses the importance of rs12913832 for prediction of eye colour and shows that the remaining five SNPs in the IrisPlex SNP set have very small effects on the outcome of the eye colour prediction in the studied population.

### 4.3. The Reporting Strategy

In this work, we tested three different eye colour categorisation systems: the quantitative system (prediction of PIE score), the two-category system (blue and brown), and the three-category system (blue, intermediate, and brown). Although it is possible to report the predicted eye colour in the form of a PIE-score, this likely requires that the end-user or the reporting laboratory translate the PIE-score into an eye colour category. Therefore, the use of a quantitative system for reporting is not relevant in a forensic genetic setting. The difference between the two- and three-category system is the definition of the intermediate eye colour, which is very difficult to perceive. When multiple individuals were asked to evaluate eye colours categorised as intermediate, they often disagreed, whereas they agreed much more frequently when eye colours were blue or brown [15]. Intermediate eye colour is predicted as the most likely eye colour (without any probability threshold) for only 8% (60 out of 729) and 14% (7.508 out of 52,488) of the possible genotype combinations in the IrisPlex Web and EC11 models, respectively [10] (Table S7). For the IrisPlex Web, the maximum probability value for prediction of intermediate eye colour is 0.62 [10]. With EC11, it is 0.94 (Table S7). In this study, we did observe intermediate eye colour predictions with EC11 with high probability values (maximum: 0.82). However, the intermediate eye colour predictions were incorrect more than half of the time (Table S6). Overall, eye colour prediction in two categories resulted in more correct predictions than eye colour prediction in three categories (92% vs. 75% for EC11; 89% vs. 72% for IrisPlex Norway and 89% vs. 72% for rs12913832) (Figure 1 and Figure 2). Hence, the definition of an intermediate eye colour category is counterintuitive, as it is both difficult to identify and predict. A recent study discusses the need for standardised methods for reporting forensic DNA phenotyping predictions to the police [25]. Reducing the complexity of eye colour predictions to only two categories results in only two hypotheses (H_1_: The person has brown eyes and H_2_: The person has blue eyes). Hence, it is possible to report the weight of the evidence with a single likelihood ratio, which resembles standard STR-profiling reports. The likelihood ratio could be supplemented with picture examples of eye colours represented by each category. This may overcome any misunderstandings or subjective opinions of eye colour interpretation, especially for eye colours that may appear non-blue and non-brown.

### 4.4. DNA Phenotyping in Forensic Genetics

The Section of Forensic Genetics in Denmark recently began offering eye colour prediction to the police using the two-category system based on the genotype of rs12913832. Prediction of EVCs can cause ethical concerns as discussed in [26]. This is especially apparent if the genetic markers used for prediction a certain trait are also linked to diseases [26]. That is not the case for rs12913832. The SNP is included in the Precision ID Ancestry Panel (Thermo Fisher Scientific, Waltham, MA, USA), which has already been validated for case work [5,27,28]. The weight of the evidence for both ancestry and eye colour predictions are reported as likelihood ratios. For eye colour predictions in the Danish population, LR = (rs12913832:GG|H_1_/rs12913832:GG|H_2_) = 0.1, LR = (rs12913832:AG|H_1_/rs12913832:AG|H_2_) = 19, and LR = (rs12913832:AA|H_1_/rs12913832:AA|H_2_) = 54 [15], where H_1_: The person has brown eyes and H_2_: The person has blue eyes. The EC11 model may be implemented at the Section of Forensic Genetics in Denmark in the future once the EC11 markers are included in a validated massively parallel sequencing (MPS) assay. The most important shortcoming of the rs12913832 two-category prediction model is the lack of information gained when including the two *OCA2* variants, rs121918166 and rs74653330. These variants were previously shown to be of importance for blue eye colour in Scandinavians with the rs12913832:AG genotype [11]. The variants had low frequencies in Danes and Swedes and were completely absent in Italians [11] (Table S3). These variants combined were ranked as second most important, with a mean variable importance of 8.5% (Table 3). In the Norwegian data set, 30 individuals with the rs12913832:AG genotype had blue eye colours according to the PIE-score. Seventeen of the 30 individuals had at least one of the *OCA2* variants and were correctly predicted to have blue eyes using the EC11 two-category model. By contrast, only one of the 30 individuals was correctly predicted with the IrisPlex Norway model, and none were correctly predicted with rs12913832. Moreover, two Norwegians with the rs12913832:AA genotype had blue eye colours according to the PIE-score. One of the two individuals was correctly predicted to have blue eye colour with the EC11 two-category model. This individual was homozygous for the rs74653330 variant. We hypothesise that the second individual has other variants in or around the *OCA2* gene that could explain the formation of blue eye colour in the rs12913832:AA genotype background. Both individuals were incorrectly predicted to have brown eye colours with the IrisPlex Norway and rs12913832 models. Lastly, 10 Norwegians with the rs12913832:GG genotype had brown eyes according to the PIE-score. Only one of them was correctly predicted to have brown eyes with the two-category EC11 and IrisPlex Norway models. This shows that we do not fully understand the formation of brown eye colour in rs12913832:GG individuals and that the EC11 model may have to be expanded further.

## Figures and Tables

**Table 1:** Eye colour variation in the variant discovery and model data sets.

	Quantitative System ^1^	Two-Category System ^2^		Three-Category System ^3^		
	Mean PIE-Score	Blue	Brown	Blue	Intermediate	Brown
Discovery data set (n = 757)	0.24	447 (59%)	310 (41%)	368 (49%)	148 (20%)	241 (31%)
Model data set (n = 523)	0.44	376 (72%)	147 (28%)	293 (56%)	123 (24%)	107 (20%)

^1.^ Statistically significant difference in mean PIE-scores between the two data sets (*p* < 0.05). ^2.^ PIE-score > 0.2: blue, and PIE-score ≤ 0.2: brown. ^3.^ PIE-score > 0.8: blue, PIE-score ≤ 0.8 to ≥−0.5: intermediate, and PIE-score < −0.5: brown.

**Table 2:** Allele frequencies of 44 variants typed in the discovery data set and 13 variants typed in the model data set.

Gene	Variant ^1^	Reference Allele	Variant Allele	Variant Allele Frequency	
				Discovery Data Set (n = 757)	Model Data Set (n = 523)
*HERC2*	rs12913832 *	**A**	**G**	**0.74**	**0.83**
*IRF4*	rs1050976	C	T	0.44	
*IRF4*	rs10530949	TCT	-	0.43	
*IRF4*	rs12211228	G	C	0.14	
*IRF4*	rs9378807	C	T	0.49	
*TYR*	rs11018509	T	A	0.29	
*TYR*	**rs1126809**	**G**	**A**	**0.24**	**0.24**
*TYR*	rs1393350 *	G	A	0.23	0.23
*TYR*	rs2047512	T	C	0.35	
*TYR*	rs34749698	T	C	0.23	
*TYR*	**rs7120151 ^2^**	**A**	**G**	**0.74**	**NA**
*TYR*	rs9919559	T	C	0.33	
*SLC24A4*	**rs10131374**	**G**	**A**	**0.14**	**0.15**
*TYRP1*	rs10491745	T	C	0.82	
*SLC24A4*	rs12590749	C	A	0.37	
*SLC24A4*	rs12880508	C	T	0.74	
*SLC24A4*	rs12894551	T	C	0.65	
*TYRP1*	**rs1408799**	**T**	**C**	**0.68**	**0.69**
*SLC24A4*	rs17128288	A	G	0.30	
*SLC24A4*	rs17128324	C	T	0.17	
*TYRP1*	rs201447946	T	TA	0.06	
*SLC24A4*	rs34755843	CGACTCT	-	0.16	
*SLC24A4*	rs35617057	G	T	0.41	
*SLC24A4*	rs4904887	C	G	0.36	
*SLC24A4*	rs4904891	G	C	0.35	
*SLC24A4*	rs4904897	C	T	0.22	
*SLC24A4*	**rs4904927**	**A**	**G**	**0.87**	**0.89**
*SLC24A4*	rs59977926	T	C	0.18	
*TYRP1*	rs62538950	A	T	0.10	
*TYRP1*	rs62538956	T	C	0.11	
*SLC24A4*	rs7144273	C	T	0.49	
*SLC24A4*	rs7152962	G	A	0.23	
*SLC24A4*	rs7401792	G	A	0.62	
*TYRP1*	rs74606098	C	T	0.06	
*TYRP1*	rs79586719	G	A	0.06	
*OCA2*	rs1800407 *	**C**	**T**	**0.07**	**0.03**
*IRF4*	rs12203592 *	C	T	0.08	0.09
*SLC24A4*	rs12896399 *	**G**	**T**	**0.49**	**0.52**
*SLC45A2*	rs16891982 *	**C**	**G**	**0.93**	**0.95**
*OCA2*	**rs1800401**	**G**	**A**	**0.04**	**0.05**
*OCA2*	rs1800414	T	C	<0.01	
*OCA2*	rs62008729	C	T	0.09	
*OCA2*	**rs121918166 ^2^**	**C**	**T**	**<0.01**	**0.01**
*OCA2*	**rs74653330 ^2^**	**C**	**T**	**<0.01**	**0.02**

^1^ Variants in bold were part of the EC11 SNPs and typed in the model data set. rs7120151 was not included in the final prediction modelling. ^2^ The combined frequency of rs121918166 and rs74653330 was 0.01 in the discovery data set. * Part of the IrisPlex prediction model [3]. NA: not analysed.

**Table 3:** Twelve variants selected for the EC11 SNP set.

Rank	Gene	Variant	Mean Variable Importance
1	*HERC2*	rs12913832 *	74.63%
2	*OCA2*	rs121918166 + rs74653330	8.54%
3	*SLC45A2*	rs16891982 *	6.23%
4	*OCA2*	rs1800407 *	5.26%
5	*TYRP1*	rs1408799	1.54%
6	*SLC24A4*	rs4904927	1.19%
7	*SLC24A4*	rs12896399 *	0.68%
8	*TYR*	rs1126809	0.47%
9	*TYR*	rs7120151 ^1^	0.46%
10	*SLC24A4*	rs10131374	0.32%
11	*OCA2*	rs1800401	0.32%

^1^ Variant not included in the final prediction modelling. * Part of the IrisPlex prediction model [3].

**Table 4:** Prediction errors for the nine eye colour prediction models (three reporting systems modelled with three SNP sets) and the IrisPlex online tool (IrisPlex Web).

Eye Colour Prediction Model	Quantitative System ^1^	Two-Category System ^2^	Three-Category System ^3^
EC11	5.07	0.26	0.59
IrisPlex Norway	5.90	0.30	0.66
rs12913832	6.96	0.32	0.69
IrisPlex Web *	NA	NA	0.80

^1^ Prediction error is the mean squared error. ^2^ Prediction error for a predicted probability, p, is log(p) if the true eye colour was blue, and log(1-p) if the true eye colour was brown. ^3^ Prediction error is the Kullback–Leibler divergence. * The IrisPlex Web predicts eye colour according to a three-category system. NA: not analysed.

**Table 5:** Sensitivity and specificity of eye colour prediction models in the two-category reporting system modelled with three SNP sets. No probability threshold was applied (pmax).

Two-Category System	Sensitivity ^1^	Specificity ^1^
rs12913832	0.92	0.84
IrisPlex Norway	0.92	0.84
EC11	0.96	0.82

^1^ Reference is blue eye colour.

**Table 6:** Sensitivity and specificity of eye colour prediction models in the three-category reporting system modelled with three SNP sets and the IrisPlex Web model. No probability threshold was applied (pmax).

Three-Category System		Sensitivity ^1^	Specificity ^1^
rs12913832	Blue	0.95	0.61
	Intermediate	0.00	1.00
	Brown	0.95	0.87
IrisPlex Norway	Blue	0.94	0.61
	Intermediate	0.10	0.97
	Brown	0.86	0.90
EC11	Blue	0.95	0.59
	Intermediate	0.15	0.95
	Brown	0.88	0.96
IrisPlex Web	Blue	0.95	0.60
	Intermediate	0.00	1.00
	Brown	0.95	0.88

^1^ Reference is blue eye colour.

## Data Availability

The data generated in the present study are included within the manuscript and its supplementary files.

## Supplementary Materials

The following are available online at https://www.mdpi.com/article/10.3390/genes12060821/s1; Table S1: Primer sequences and primer concentrations for 24 plex and 11 plex typed in the variant discovery data set; Table S2: Primer sequences and primer concentrations for 14 plex typed with SNaPshot in the model data set; Table S3: Allele frequencies of 44 variants typed in the discovery data set comprising Danish, Swedish, and Italian individuals (DK/SWE/ITA) and 13 variants typed in the model data set comprising Norwegian individuals (NO); Table S4: Mean variable importance of top 20 variants; Table S5: Confusion matrices for two-category system prediction models; one for each SNP set and each threshold (pmax and p > 0.7); Table S6: Confusion matrices for three-category system prediction models; one for each SNP set and each threshold (pmax, p > 0.5, and p > 0.7); Table S7: Eye colour prediction outcomes for 52,488 possible genotype combinations using the EC11 three-category model.
