# Supplementary material for: Prediction of Eye Colour in Scandinavians Using the EyeColour 11 (EC11) SNP Set
Source: Genes (Basel). 2021 May 27;12(6):821. doi: 10.3390/genes12060821 (PMC8227851; doi:10.3390/genes12060821)
Supplement: Supplementary file 1 [file genes-12-00821-s001.zip › genes-1238438-SI/Supp.material/S3table.pdf]

Table S3: Allele frequencies of 44 variants typed in the discovery data set comprising Danish, Swedish, and Italian individuals (DK/SWE/ITA) and 13 variants typed in the model data set comprising Norwegian individuals (NO).

				**Variant allele frequency**				
**Gene**	**Variant[1]**	**Reference allele**	**Variant allele**	**DK (n=357)**	**SWE (n=182)**	**ITA (n=218)**	**DK-SWE-ITA (n=757)**	**NO (n=523)**
HERC2	**rs12913832***	**A**	**G**	**0.85**	**0.84**	**0.48**	**0.74**	**0.83**
IRF4	rs1050976	C	T	0.42	0.45	0.46	0.44	
IRF4	rs10530949	TCT	-	0.45	0.41	0.42	0.43	
IRF4	rs12211228	G	C	0.15	0.13	0.14	0.14	
IRF4	rs9378807	C	T	0.47	0.48	0.51	0.49	
TYR	rs11018509	T	A	0.27	0.34	0.28	0.29	
TYR	**rs1126809**	**G**	**A**	**0.23**	**0.26**	**0.25**	**0.24**	**0.24**
TYR	rs1393350*	G	A	0.22	0.25	0.23	0.23	0.23
TYR	rs2047512	T	C	0.38	0.37	0.30	0.35	
TYR	rs34749698	T	C	0.22	0.25	0.23	0.23	
TYR	**rs7120151**	**A**	**G**	**0.75**	**0.70**	**0.76**	**0.74**	***NA***
TYR	rs9919559	T	C	0.31	0.38	0.33	0.33	
SLC24A4	**rs10131374**	**G**	**A**	**0.15**	**0.10**	**0.16**	**0.14**	**0.15**
TYRP1	rs10491745	T	C	0.81	0.83	0.82	0.82	
SLC24A4	rs12590749	C	A	0.33	0.34	0.46	0.37	
SLC24A4	rs12880508	C	T	0.76	0.75	0.70	0.74	
SLC24A4	rs12894551	T	C	0.64	0.63	0.69	0.65	

TYRP1	**rs1408799**	**T**	**C**	**0.70**	**0.68**	**0.64**	**0.68**	**0.69**
SLC24A4	rs17128288	A	G	0.32	0.29	0.27	0.30	
SLC24A4	rs17128324	C	T	0.15	0.15	0.22	0.17	
TYRP1	rs201447946	T	TA	0.06	0.06	0.06	0.06	
SLC24A4	rs34755843	CGACTCT	-	0.17	0.16	0.15	0.16	
SLC24A4	rs35617057	G	T	0.41	0.44	0.40	0.41	
SLC24A4	rs4904887	C	G	0.33	0.33	0.42	0.36	
SLC24A4	rs4904891	G	C	0.37	0.36	0.30	0.35	
SLC24A4	rs4904897	C	T	0.24	0.22	0.20	0.22	
SLC24A4	**rs4904927**	**A**	**G**	**0.90**	**0.89**	**0.81**	**0.87**	**0.89**
SLC24A4	rs59977926	T	C	0.17	0.18	0.20	0.18	
TYRP1	rs62538950	A	T	0.10	0.09	0.12	0.10	
TYRP1	rs62538956	T	C	0.12	0.11	0.10	0.11	
SLC24A4	rs7144273	C	T	0.50	0.50	0.46	0.49	
SLC24A4	rs7152962	G	A	0.23	0.20	0.28	0.23	
SLC24A4	rs7401792	G	A	0.64	0.66	0.56	0.62	
TYRP1	rs74606098	C	T	0.06	0.07	0.06	0.06	
TYRP1	rs79586719	G	A	0.07	0.07	0.06	0.06	
OCA2	**rs1800407***	**C**	**T**	**0.04**	**0.05**	**0.11**	**0.07**	**0.03**
IRF4	rs12203592*	C	T	0.07	0.06	0.13	0.08	0.09
SLC24A4	**rs12896399***	**G**	**T**	**0.56**	**0.51**	**0.36**	**0.49**	**0.52**
SLC45A2	**rs16891982***	**C**	**G**	**0.96**	**0.95**	**0.87**	**0.93**	**0.95**
OCA2	**rs1800401**	**G**	**A**	**0.04**	**0.01**	**0.07**	**0.04**	**0.05**
OCA2	rs1800414	T	C	0.00	0.01	0.00	<0.01	
OCA2	rs62008729	C	T	0.11	0.10	0.04	0.09	

OCA2	**rs121918166[2]**	**C**	**T**	**0.01**	**0.01**	**0.00**	**<0.01**	**0.01**
OCA2	**rs74653330[2]**	**C**	**T**	**0.00**	**0.01**	**0.00**	**<0.01**	**0.02**

[1]Variants in bold were part of the EC11 SNPs and typed in the model data set. rs7120151 was not included in the final prediction modelling

[2]The combined frequency of rs121918166 and rs74653330 was 0.01 in the discovery data set

*Part of IrisPlex prediction model [3]

NA: Not analysed
