# Supplementary material for: Prediction of Eye Colour in Scandinavians Using the EyeColour 11 (EC11) SNP Set
Source: Genes (Basel). 2021 May 27;12(6):821. doi: 10.3390/genes12060821 (PMC8227851; doi:10.3390/genes12060821)
Supplement: Supplementary file 1 [file genes-12-00821-s001.zip › genes-1238438-SI/Supp.material/S4table.pdf]

S4 Table: Mean variable importance of top 20 variants.

Rank	Gene	Variant	Mean variable importance
1	*HERC2*	rs12913832*	74.63%
2	*OCA2*	rs121918166 + rs74653330	8.54%
3	*SLC45A2*	rs16891982*	6.23%
4	*OCA2*	rs1800407*	5.26%
5	*TYRP1*	rs1408799	1.54%
6	*SLC24A4*	rs4904927	1.19%
7	*SLC24A4*	rs12896399*	0.68%
8	*TYR*	rs1126809	0.47%
9	*TYR*	rs7120151[1]	0.46%
10	*SLC224A4*	rs10131374	0.32%
11	*OCA2*	rs1800401	0.32%
12	*SLC24A4*	rs17128324	0.11%
13	*TYRP1*	rs10491745	0.066%
14	*TYRP1*	rs62538956	0.055%
15	*TYRP1*	rs74606098	0.048%
16	*TYR*	rs2047512	0.044%
17	*TYR*	rs11018509	0.015%
18	*TYR*	rs1393350*	0.012%
19	*TYRP1*	rs201447946	0.007%
20	*IRF4*	rs1050976	0.006%

[1]Variant not included in final EC11 SNP set

*Part of IrisPlex prediction model [3]
