# Supplementary material for: Prediction of Eye Colour in Scandinavians Using the EyeColour 11 (EC11) SNP Set
Source: Genes (Basel). 2021 May 27;12(6):821. doi: 10.3390/genes12060821 (PMC8227851; doi:10.3390/genes12060821)
Supplement: Supplementary file 1 [file genes-12-00821-s001.zip › genes-1238438-SI/Supp.material/S7table.pdf]

Table S7: Eye colour prediction outcomes for 52,488 possible genotype combinations using the EC11 three-category model.

	**Blue**	**Brown**	**Intermediate**
Number of predictions[1]	40,054	4,926	7,508
Maximum probability value	0.999	1.00	0.941
Minimum probability value	0.341	0.348	0.337

[1]Predictions of 52,488 possible genotype combinations in the EC11 SNP set using the three-category system. The predicted eye colour was the eye colour category with the highest probability (pmax). For comparison, only 226 genotype combinations were observed in the model data set
